# Examining Self-Compassion and Self-Leadership as Predictors of Job Satisfaction, Psychological Health, and Turnover Intention in Midwives Across Demographic Factors

**DOI:** 10.3390/healthcare14070873

**Published:** 2026-03-28

**Authors:** Filiz Okumuş, İmran Aslan

**Affiliations:** 1 Midwifery Department, Faculty of Health Sciences, Istanbul Atlas University, Istanbul 34403, Türkiye; 2 Health Management Department, Faculty of Health Sciences, Bingöl University, Bingöl 12000, Türkiye; imranaslan@gmail.com

**Keywords:** self-leadership, self-compassion, midwifery, workforce sustainability, job satisfaction, turnover intention, job performance, organizational factors, spiritual awareness, Turkey

## Abstract

**Highlights:**

**What are the main findings?**

- Self-leadership, particularly natural reward strategies emphasizing intrinsic motivation, meaning, and purpose in professional roles, emerged as a strong predictor of midwives’ job performance, reflecting the role of inner resources aligned with spiritual awareness in healthcare practice.
- In contrast, job satisfaction and turnover intention were predominantly shaped by organizational and structural conditions, indicating that individual psychological and spiritual capacities alone are insufficient to sustain well-being and retention in unsupportive work environments.

**What are the implications of the main findings?**

- Promoting spiritually aware, humanized, and holistic care in midwifery requires multi-level strategies that integrate the development of inner capacities (such as self-leadership and meaning-oriented engagement) with organizational reforms that support ethical, compassionate, and sustainable practice.
- Self-leadership-based approaches may enhance professional performance and purposeful engagement, improving job satisfaction and reducing turnover necessitate organizational-level interventions that address working conditions, institutional support, and structural constraints on humanized care.

**Abstract:**

**Background/Objectives:** Midwifery workforce sustainability faces critical challenges including high burnout and turnover rates threating the service quality and the maternal health outcomes. While self-leadership and self-compassion represent promising psychological resources, their roles relative to organizational factors remain underexplored. This study examined associations between self-leadership, self-compassion, and workforce outcomes (job satisfaction, turnover intention, performance) among Turkish midwives. **Methods**: A cross-sectional survey was conducted with 346 midwives working in diverse healthcare settings across Turkey from May 2021 to April 2022. Data were collected through an online self-report questionnaire using validated scales for self-leadership and self-compassion as well as measures of job satisfaction, turnover intention, and job performance, and including demographic and organizational items. Descriptive statistics, one-way ANOVA (with Eta-squared [*η*^2^] calculated to determine effect size), and correlation analyses were conducted, followed by hierarchical multiple regression and binary logistic regression to examine predictive relationships, with organizational factors entered before psychological resources. **Results:** Self-leadership and self-compassion demonstrated a moderate positive correlation (r = 0.342, *p* < 0.01). Self-leadership strongly predicted job performance (OR = 2.497, *p* = 0.001), particularly through natural reward strategies emphasizing intrinsic motivation (OR = 1.970, *p* < 0.001). However, neither psychological resource significantly predicted job satisfaction or turnover intention when organizational factors were included. Work schedule, healthcare setting, professional position, and income emerged as primary predictors of satisfaction and retention. Work experience predicted increased psychological distress (OR = 1.073, *p* = 0.003). **Conclusions:** Psychological resources demonstrate domain-specific effects on workforce outcomes in midwifery: self-leadership strategies strongly enhance job performance, whereas job satisfaction and turnover intention are influenced primarily by organizational conditions. These findings highlight the need for multi-level strategies to support the sustainability of the midwifery workforce.

## 1. Introduction

### 1.1. Background

Healthcare organizations are increasingly characterized by complexity, high labor intensity, and growing demands for collaboration, adaptability, and resilience [1,2]. Rapid technological developments, more complex patient profiles, and intensified regulatory pressures have challenged traditional hierarchical leadership models in healthcare systems worldwide. In response, leadership in healthcare has evolved toward more participatory and distributed approaches that recognize the professional agency, autonomy, and expertise of healthcare workers across disciplines [3,4]. These developments also highlight the growing importance of spirituality as a source of meaning, ethical grounding, and inner resilience that supports compassionate care, wellbeing, and sustained engagement among healthcare professionals [5,6]. These contemporary perspectives emphasize that leadership is not limited to formal managerial roles but is embedded in everyday clinical practice [7,8].

This transformation is particularly evident in healthcare professions that require continuity of care, autonomous decision-making, and intensive interpersonal interaction. Within this context, midwifery represents a field in which clinical responsibility is high and professional leadership plays a crucial role in ensuring the quality and safety of care [9,10]. Within midwifery, leadership extends beyond managerial function to constitute a core professional competency directly linked to clinical autonomy, safe childbirth practices, and respectful maternity care [7,11]. This form of leadership is increasingly understood to incorporate workplace spirituality, reflected in a sense of meaning, compassion, and value-driven practice that supports respectful relationships with women, families, and interdisciplinary teams. International evidence highlights substantial disparities in midwifery leadership structures and their implications for maternal and neonatal outcomes. While countries with strong governance frameworks supporting midwifery autonomy and spiritually grounded, values-based leadership report favorable birth outcomes and higher professional satisfaction, contexts lacking such structures demonstrate poorer indicators of maternal and newborn health [12,13].

Beyond clinical outcomes, leadership in midwifery carries broader implications for health system sustainability and population health. Midwives accompany women during critical life transitions, and their professional effectiveness influences not only immediate birth outcomes but also long-term family wellbeing [14,15]. Developing leadership competencies among midwives strengthens advocacy, enhances professional confidence, and supports adaptive responses to organizational change [9]. Despite these potential benefits, midwives continue to face substantial workplace challenges that threaten their capacity to provide high-quality care and sustain their careers.

In this context, midwifery globally faces a workforce crisis marked by declining job satisfaction, rising turnover intention, and growing concerns about sustainability [1,10]. Among midwives, job satisfaction is shaped by workload, professional autonomy, organizational support, and interpersonal relationships [16]. Turnover intention—a strong predictor of actual turnover—imposes substantial organizational costs and threatens continuity of care [17,18]. Job performance, another critical workforce outcome, directly affects care quality and patient safety [19]. Beyond these workforce outcomes, psychological health remains under-addressed in midwifery practice. Evidence consistently reports high levels of burnout, emotional exhaustion, anxiety, and secondary traumatic stress among midwives. Evidence consistently reports high levels of burnout, emotional exhaustion, anxiety, and secondary traumatic stress among midwives [6,20]. Exposure to traumatic births, responsibility for maternal and neonatal outcomes, medico-legal pressures, and sustained emotional labor contribute to this strain Paradoxically, accumulating professional experience is associated with emotional detachment and compassion fatigue, raising concerns about long-term professional sustainability [6,21].

### 1.2. Literature Review

#### 1.2.1. Self-Leadership in Midwifery

Traditional hierarchical leadership models are increasingly insufficient for professions such as midwifery requiring high autonomy, rapid decision-making, and adaptive problem-solving in unpredictable clinical environments [22,23]. In this context, self-leadership has emerged as a foundational framework describing how individuals regulate their thoughts, emotions, and behaviors to achieve professional goals and sustain ethical practice. It also includes the use of spiritual resources such as meaning, values, and purpose to maintain resilience in demanding clinical settings [24,25].

Self-leadership theory conceptualizes leadership as beginning with the self, emphasizing internal self-influence processes that precede and support leadership of others [25]. The framework comprises three core strategy categories: behavior-focused strategies, natural reward strategies, and constructive thought pattern strategies [24]. Empirical studies within healthcare settings associate self-leadership with improved job performance, innovation, problem-solving, and psychological wellbeing [26,27]. For midwives, self-leadership supports effective management of emotional labor, autonomous clinical decision-making, and collaborative practice [19].

#### 1.2.2. Self-Compassion as a Complementary Resource

While self-leadership emphasizes goal attainment and self-regulation, self-compassion offers a complementary psychological resource focused on self-care, emotional balance, and sustainability [24,28,29]. Self-compassion involves responding to personal failure or distress with kindness, recognition of shared human experience, and mindful awareness rather than self-criticism [29,30].

Within healthcare professions, higher self-compassion is associated with lower burnout, reduced secondary traumatic stress, and greater resilience [31]. Given the emotionally demanding nature of midwifery practice, self-compassion may play a protective role in maintaining empathic engagement and psychological wellbeing [6]. However, studies indicate that self-compassion levels among healthcare professionals often remain suboptimal, potentially reflecting professional cultures that prioritize endurance and self-sacrifice over self-care [31,32].

#### 1.2.3. Research Gap and Study Rationale

Despite increasing recognition of self-leadership and self-compassion as valuable psychological resources, several gaps persist in the literature. First, these constructs have rarely been examined simultaneously within the same midwifery sample, limiting understanding of their relative and combined contributions to workforce outcomes. Second, leadership research continues to underrepresent midwifery, focusing predominantly on nursing or medical professionals. Third, the interaction between individual psychological resources and organizational factors in predicting job satisfaction, turnover intention, and performance remains insufficiently explored. Finally, potential moderating effects of demographic variables such as age, experience, and family responsibilities require further investigation.

### 1.3. Study Objectives and Hypotheses

The present study investigates self-leadership and self-compassion as key psychological resources among practicing midwives and examines how these resources relate to critical workforce outcomes. Specifically, the study aims (i) to determine the association between self-leadership and self-compassion, (ii) to examine whether self-leadership and self-compassion levels differ according to demographic and organizational characteristics, and (iii) to test the predictive roles of self-leadership and self-compassion in explaining job satisfaction, turnover intention, and job performance. In addition, the study evaluates the extent to which organizational factors contribute to turnover intention beyond individual psychological resources and explores the potential moderating influence of demographic variables on the associations between psychological resources and professional outcomes.

Accordingly, the following hypotheses were formulated:

**Hypothesis** **1.** *Self-leadership and self-compassion will be significantly and positively associated.*

**Hypothesis** **2.** *Self-leadership and self-compassion levels will differ significantly according to selected demographic and organizational characteristics (e.g., age, work experience, work schedule, and healthcare setting).*

**Hypothesis** **3.** *Self-leadership and self-compassion will significantly predict key workforce outcomes, such that they will positively predict job satisfaction (H3a), negatively predict turnover intention (H3b), and positively predict job performance (H3c).*

## 2. Materials and Methods

### 2.1. Design, Research Field, and Participants

This study employed a cross-sectional, descriptive-correlational design to examine relationships between self-leadership, self-compassion, and key workforce outcomes among practicing midwives. The study was conducted with midwives working across four practice settings within the Türkiye healthcare system: public hospitals (58.7% of the sample), private hospitals (19.7%), family health centers (17.1%), and university hospitals (4.6%). This distribution reflects the predominance of public sector employment among Turkish midwives while capturing a range of clinical contexts.

Participants were eligible if they were currently employed as registered midwives in Türkiye, actively engaged in clinical practice, possessed sufficient Turkish language proficiency, and provided informed consent. Exclusion criteria included having less than one year of professional experience, not belonging to the target professional group, not being actively engaged in clinical practice during the study period, being a midwifery student, holding exclusively non-clinical roles, and submitting incomplete survey responses. Midwives with less than one year of experience were excluded to ensure that participants had sufficient exposure to workplace conditions and organizational processes relevant to job satisfaction, turnover intention, and performance.

The study was conducted in accordance with the Declaration of Helsinki. Ethical approval was obtained from the Institutional Review Board prior to participant recruitment (Approval Number: 74791132-604.01.01-971; Date: 5 April 2021).

### 2.2. Recruitment and Sample Size

A convenience sampling strategy was employed. Participants were recruited through professional midwifery association networks, direct outreach to healthcare institutions with requests to distribute the survey among employed midwives, social media announcements in professional groups, and participant referrals.

Sample size was determined through an a priori power analysis conducted using G*Power 3.1 (Heinrich Heine University Düsseldorf, Germany),assuming a medium effect size (r ≈ 0.30), a 95% confidence level (α = 0.05), and 80% statistical power (1 − β = 0.80). The analysis indicated that a minimum of 84 participants would be required to detect correlations of this magnitude, while 130–170 participants would be sufficient for multivariable regression analyses depending on the number of predictors included. Based on these estimates, the target sample size was set to exceed the minimum requirement for multivariable analyses.

**Figure 1:**
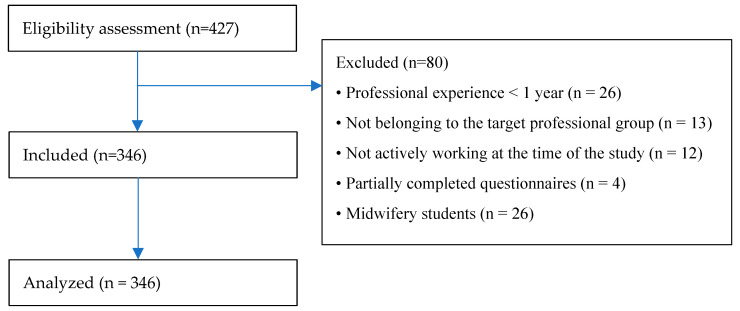
STROBE flow chart.

The final sample consisted of 346 midwives (N = 346), exceeding the calculated requirements and providing sufficient statistical power for all planned analyses. Figure 1 summarizes the number of participants assessed during the study inclusion process.

### 2.3. Procedures

Following institutional ethical approval, recruitment commenced through the channels described above. Data were collected using an online survey administered via Google Forms (Google LLC, Mountain View, CA, USA). The survey landing page provided detailed study information, and participants gave electronic informed consent prior to accessing the questionnaire. The online survey was configured to require mandatory responses and to limit multiple submissions. Exclusions were based on predefined eligibility criteria and data quality assessments.

Participation was anonymous, and data confidentiality was ensured through secure data handling procedures. Measures were implemented to prevent duplicate submissions, and responses to primary study variables were required to enhance data quality. Data collection occurred over a 12-month period from May 2021 to April 2022. The average survey completion time was approximately 20–25 min.

#### 2.3.1. Revised Self-Leadership Scale (RSLQ)

Self-leadership was assessed using the Revised Self-Leadership Questionnaire (RSLQ), developed by Houghton and Neck (2002) [24] based on Manz’s (1986) [25] self-leadership theory and adapted into Turkish by Tabak et al. (2013) [33]. The Turkish version consists of 29 items covering three strategic dimensions: behavior-focused strategies, natural reward strategies, and constructive thought pattern strategies.

Items are rated on a 5-point Likert scale ranging from 1 (strongly disagree) to 5 (strongly agree), with higher scores indicating greater self-leadership. The Turkish adaptation demonstrated acceptable reliability (Cronbach’s α = 0.77) [33]. In the present study, internal consistency was excellent (Cronbach’s α = 0.90).

#### 2.3.2. Self-Compassion Scale

Self-compassion was measured using the Self-Compassion Scale (SCS) developed by Neff (2003) [34]. The scale comprises 26 items assessing self-kindness, common humanity, and mindfulness, along with their negative counterparts. Items are rated on a 5-point Likert scale ranging from 1 (almost never) to 5 (almost always) [34].

The Turkish adaptation by Deniz et al. (2008) [35] confirmed a unidimensional structure, with two items removed due to low item–total correlations, resulting in a 24-item scale. The Turkish version demonstrated strong psychometric properties (Cronbach’s α = 0.89; test–retest reliability = 0.83) [35]. In the present study, internal consistency was good (Cronbach’s α = 0.667).

#### 2.3.3. Job Satisfaction Measure

Job satisfaction was assessed using a single-item self-report measure rating overall work satisfaction on a scale from 1 (not at all satisfied) to 10 (extremely satisfied). Single-item job satisfaction measures demonstrate adequate validity and reliability, showing strong correlations with multi-item scales while minimizing respondent burden [36].

#### 2.3.4. Turnover Intention Measure

Turnover intention was assessed using a direct single-item question: “Are you currently considering leaving your current position?” with dichotomous response options (Yes/No). Participants responding affirmatively were classified as having present turnover intention. This straightforward approach aligns with methodologies emphasizing brevity and ease of administration in high-pressure healthcare settings [37]. Furthermore, given that the definition and operational measurement of turnover intention vary widely across research studies [38], this direct assessment provides a clear and immediate indicator of potential workforce loss.

#### 2.3.5. Job Performance Measure

Job performance was assessed through self-report using a single item asking participants to rate their overall job performance as “good,” “moderate,” or “poor.” Similarly, in studies conducted on healthcare workers [6,19], single-item and rating-based (e.g., good/moderate/poor) self-report measures have been used to assess performance indicators such as job satisfaction or academic achievement, and comparisons between groups were made in the analyses. Although self-report measures have certain limitations, they are noted to offer valuable insights into perceived professional effectiveness and are associated with objective evaluations [39].

#### 2.3.6. Demographic and Professional Questionnaire

A comprehensive questionnaire assessed: age, work experience, educational level, income status, healthcare setting, position, work schedule, professional association membership, number of children, marital status, and history of mental health diagnosis.

### 2.4. Data Analysis

All analyses were conducted using IBM SPSS Statistics Version 26.0 (IBM Corp., Armonk, NY, USA). Descriptive statistics were calculated using means and standard deviations for continuous variables and frequencies and percentages for categorical variables. Normality was assessed using skewness and kurtosis values. Given the observational, cross-sectional design, analyses focused on identifying associations rather than inferring causality. Pearson correlation analyses were used to examine relationships among continuous variables. Group differences were analyzed using independent samples *t*-tests and one-way ANOVA. Multiple regression analyses were conducted to evaluate the predictive roles of self-leadership and self-compassion on job satisfaction, turnover intention, and job performance. Internal consistency of multi-item scales was assessed using Cronbach’s alpha. Statistical significance was set at α = 0.05, and effect sizes were reported alongside *p*-values.

## 3. Results

### 3.1. Participant Characteristics

A total of 346 registered midwives practicing across diverse healthcare settings in Turkey participated in this study. The sample’s mean age was 32.42 years (SD = 7.81, range = 22–55 years). Work schedule distribution revealed that 43.6% worked permanent day shifts, 41.3% worked rotating shifts (mixed day and night), and 15.0% worked permanent night shifts. Healthcare setting analysis showed that 58.7% of participants were employed in public hospitals, 19.7% in private hospitals, 17.1% in family health centers, and 4.6% in university hospitals. With respect to professional position, 81.5% worked as clinical staff, 13.6% served as team leaders, and 4.9% held managerial positions. Professional association membership was present among 37.0% of participants, while 63.0% reported no membership affiliation (Table 1).

Participants reported generally high levels of work satisfaction, with a mean self-rated satisfaction score of 8.26 (SD = 1.90) on a 10-point scale. Regarding mental health, 5.5% of participants reported having received a diagnosis of a mental health condition. When asked to rate their job performance, 83.5% characterized their performance as good, while 16.5% rated it as moderate. Notably, 31.5% of participants indicated present turnover intention, representing nearly one-third of the sample. Additionally, 83.5% of participants reported that the midwifery profession contributed to their personal development, suggesting overall positive professional identity despite the substantial proportion expressing turnover intention.

### 3.2. Descriptive Statistics for Main Study Variables

Descriptive statistics for all primary study variables, including means, standard deviations, ranges, skewness, kurtosis, and internal consistency reliability coefficients, are presented in Table 2. Participants demonstrated high levels of overall self-leadership (M = 4.06, SD = 0.49, range = 1.93–5.00), indicating frequent use of self-leadership strategies. In contrast, levels of self-compassion—conceptualized in this context as a core spiritual and emotional resource—were moderate (M = 3.25, SD = 0.39, range = 2.17–5.00). All variables demonstrated acceptable distributional properties, with skewness and kurtosis values falling within acceptable ranges (between −2 and +2), supporting the appropriateness of parametric statistical procedures.

Examination of self-leadership subdimensions revealed that participants scored highest on constructive thinking strategies (M = 4.20, SD = 0.56) and natural reward strategies (M = 4.29, SD = 0.70), while behavior-focused strategies showed somewhat lower but still above-midpoint scores (M = 3.87, SD = 0.53). Within constructive thinking strategies, “evaluating thoughts and ideas” achieved the highest mean score (M = 4.30, SD = 0.58), followed closely by “imagining successful performances” (M = 4.24, SD = 0.68) and “self-talk” (M = 4.06, SD = 0.76). The natural reward strategies dimension, represented by “thinking about natural rewards,” also demonstrated high utilization (M = 4.29, SD = 0.70).

Among behavior-focused strategies, “self-monitoring” achieved the highest score (M = 4.29, SD = 0.59), followed by “self-rewarding” (M = 3.95, SD = 0.95) and “reminders” (M = 3.85, SD = 1.04). The lowest mean score was observed for “self-punishment” (M = 3.37, SD = 0.88), suggesting that participants employed punitive self-regulatory strategies less frequently than other self-leadership approaches. This pattern indicates that midwives in this sample preferentially utilized constructive and supportive self-leadership strategies—such as cognitive reframing and focus on intrinsic rewards—over punitive behavioral strategies.

Regarding knowledge sources for self-leadership and self-compassion concepts, 37.0% of participants reported having received no information about self-leadership from any source, while 28.3% had learned through formal educational activities, 20.5% through visual media, and 8.4% through printed materials. For self-compassion, 46.8% reported no prior exposure to the concept, 34.7% had learned through formal education, 16.5% through visual media, and 7.8% through printed materials. These findings suggest that structured education on both self-leadership and self-compassion remains limited among practicing midwives in Turkey.

### 3.3. Bivariate Correlations Among Study Variables

**Figure 2:**
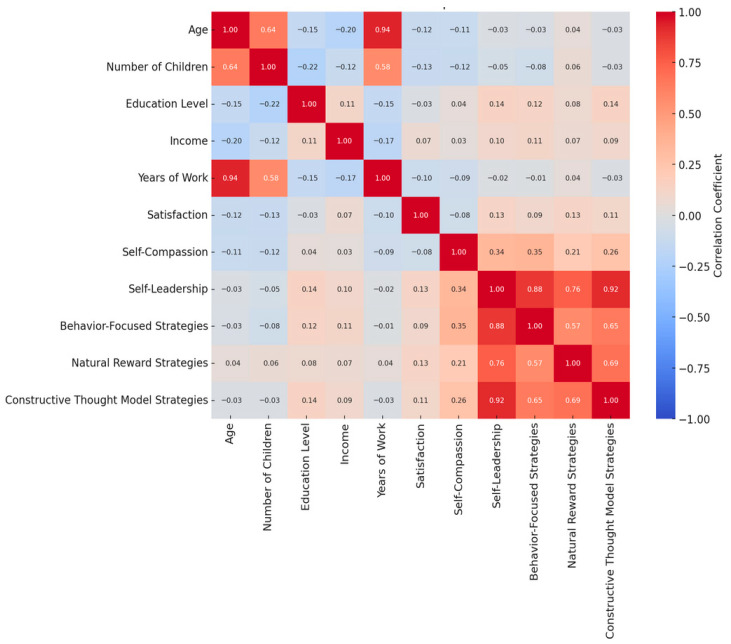
Correlation map.

Pearson correlation coefficients among study variables are presented in Figure 2. Consistent with Hypothesis 1, a statistically significant positive correlation of moderate magnitude was observed between self-leadership and self-compassion (*r* = 0.342, *p* < 0.01), indicating that midwives who reported higher self-leadership also tended to report higher self-compassion, reflecting a concurrent use of behavioral strategies and inner spiritual awareness. Among self-leadership subdimensions, behavior-focused strategies demonstrated the strongest association with self-compassion (*r* = 0.355, *p* < 0.01), followed by natural reward strategies and constructive thinking strategies.

Examination of correlations among self-leadership subdimensions revealed very strong positive associations, with the correlation between overall self-leadership and constructive thinking strategies approaching *r* = 0.92, indicating substantial overlap among these self-regulatory approaches. Self-leadership subdimensions (behavior-focused, natural reward, and constructive thinking strategies) demonstrated intercorrelations ranging from moderate to very strong (*r* values from 0.60 to 0.92), consistent with their conceptualization as interrelated components of a broader self-leadership construct.

Correlations between demographic variables and primary study variables revealed several notable patterns. Age demonstrated a strong positive correlation with number of children (*r* = 0.644, *p* < 0.01), as expected. However, age showed significant negative correlations with income level (*r* = −0.204, *p* < 0.01) and educational level (*r* = −0.150, *p* < 0.01), suggesting that younger midwives in this sample tended to have higher educational credentials and reported higher income levels. Both age (*r* = −0.118, *p* < 0.05) and number of children (*r* = −0.129, *p* < 0.05) demonstrated weak but statistically significant negative correlations with job satisfaction, indicating that job satisfaction tended to decline slightly with increasing age and family responsibilities.

Correlations between psychological resources (self-leadership and self-compassion) and workforce outcomes were generally weak to moderate in magnitude. Self-leadership demonstrated small positive correlations with job satisfaction (*r* values ranging from 0.10 to 0.25 across subdimensions), while self-compassion showed similarly modest associations. These relatively weak correlations, while statistically significant in some cases, suggested that the relationships between individual psychological resources and workforce outcomes might be more complex than simple bivariate associations would indicate.

### 3.4. Demographic and Organizational Differences in Self-Leadership and Self-Compassion

One-way analysis of variance (ANOVA) was conducted to test Hypothesis 2, examining whether self-leadership and self-compassion levels differed according to demographic and organizational characteristics. Results are presented in Table 3. Self-compassion levels did not differ significantly across any demographic or organizational variables examined (all *p* > 0.05), including educational level, income status, healthcare setting, work schedule, and position. This finding indicates that self-compassion levels were relatively uniform across different demographic and professional subgroups in this sample, suggesting that this inner spiritual capacity operates independently of structural or positional factors.

In contrast, self-leadership demonstrated significant variation according to educational level (*F*(2, 343) = 5.125, *p* = 0.006; *η*^2^ = 0.0290) with a small to medium effect size and marginally significant variation by position (*F*(2, 343) = 2.762, *p* = 0.065; *η*^2^ = 0.016) with a small effect size. Further examination of self-leadership subdimensions revealed more specific patterns. Behavior-focused strategies differed significantly by educational level (*F*(2, 343) = 3.250, *p* = 0.040; *η*^2^ = 0.0184) with a small effect size and higher educated midwives reporting greater use of these strategies. Natural reward strategies showed significant differences by both educational level (*F*(2, 343) = 6.030, *p* = 0.003; *η*^2^ = 0.0340) with a small to medium effect size and position (*F*(2, 343) = 5.297, *p* = 0.005; *η*^2^ = 0.0300) with a small to medium effect size and master’s degree holders and those in leadership positions reporting higher scores. Similarly, constructive thinking strategies varied significantly by educational level (*F*(2, 343) = 4.934, *p* = 0.008; *η*^2^ = 0.028) with a small effect size and position (*F*(2, 343) = 3.119, *p* = 0.045; *η*^2^ = 0.0178) a small effect size.

No significant differences in self-leadership or self-compassion were observed across income levels, healthcare settings, or work schedules (all *p* > 0.05). Overall, these findings provide partial support for Hypothesis 2, demonstrating that educational level and professional position influence self-leadership but not self-compassion, while other demographic and organizational factors showed limited association with either psychological resource.

### 3.5. Predictors of Job Satisfaction

Hierarchical multiple regression analysis was conducted to examine predictors of job satisfaction, testing Hypothesis 3a regarding the predictive roles of self-leadership and self-compassion. Variables were entered in three blocks: Block 1 included demographic controls (age, marital status, number of children, educational level, work experience), Block 2 added organizational factors (income status, healthcare setting, work schedule, position), and Block 3 added psychological resources (self-compassion and self-leadership).

Results revealed that organizational factors emerged as the primary significant predictors of job satisfaction, while psychological resources did not demonstrate significant independent contributions. Specifically, work schedule (*B* = 0.431, *β* = 0.209, *t* = 3.872, *p* < 0.001), healthcare setting (*B* = 0.413, *β* = 0.199, *t* = 3.688, *p* < 0.001), and position (*B* = 0.551, *β* = 0.153, *t* = 2.891, *p* = 0.004) each demonstrated significant positive associations with job satisfaction. Midwives working in certain schedule configurations, specific healthcare settings, and higher organizational positions reported significantly higher job satisfaction.

Contrary to expectations, neither self-leadership nor self-compassion emerged as significant predictors of job satisfaction when organizational factors were included in the model (both *p* > 0.05). This finding indicates that while organizational and structural work characteristics strongly influenced job satisfaction, individual psychological resources did not explain additional variance beyond these contextual factors. Hypothesis 3a was therefore not supported with respect to psychological resources, although organizational predictors demonstrated the expected associations with job satisfaction.

The final model explained approximately 18% of variance in job satisfaction (*R*^2^ = 0.18, *F*(12. 333) = 6.11, *p* < 0.001), with organizational factors accounting for the majority of explained variance. These findings suggest that job satisfaction among midwives is more strongly determined by external work conditions than by internal psychological resources.

### 3.6. Predictors of Turnover Intention

Hierarchical binary logistic regression analysis was conducted to identify predictors of turnover intention (present vs. absent), testing Hypothesis 3b. Variables were entered using forward stepwise (conditional) method to identify the most parsimonious set of significant predictors from among demographic variables (age, marital status, number of children, educational level, work experience), organizational factors (income status, healthcare setting, work schedule, position, job satisfaction), psychological resources (self-compassion, self-leadership), and job performance.

The final model retained three significant predictors: income status, work schedule, and job performance (Table 4). Income status demonstrated an overall significant effect on turnover intention (χ^2^(2) = 10.357, *p* = 0.006), although individual income category coefficients did not reach conventional significance levels (income less than expenses: *B* = −0.496, *p* = 0.081, *OR* = 0.609; income equal to expenses: *B* = 0.634, *p* = 0.129, *OR* = 1.885). This pattern suggests that income influences turnover intention through its overall distribution rather than through specific category comparisons.

Work schedule emerged as a significant predictor (χ^2^(2) = 7.469, *p* = 0.024), with midwives working permanent day shifts demonstrating 2.3 times higher odds of turnover intention compared to the reference category (*B* = 0.843, *p* = 0.016, *OR* = 2.323, 95% CI [1.162, 4.644]), and those working permanent night shifts showing 1.8 times higher odds (*B* = 0.572, *p* = 0.030, *OR* = 1.772, 95% CI [1.056, 2.973]). Job performance demonstrated a significant protective effect, with each unit increase in performance rating associated with approximately 49% lower odds of turnover intention (*B* = −0.669, *p* = 0.029, OR = 0.512, 95% CI [0.281, 0.932]).

Critically, neither self-leadership nor self-compassion entered the final model as significant predictors of turnover intention when organizational factors and job performance were considered (both *p* > 0.05 in preliminary models). This finding indicates that turnover intention is primarily driven by organizational conditions and performance perceptions rather than by individual psychological resources. Hypothesis 3b was therefore not supported regarding the predictive roles of self-leadership and self-compassion.

The final model demonstrated acceptable fit (Hosmer–Lemeshow χ^2^(8) = 6.432, *p* = 0.598) and correctly classified 69.9% of cases, with Nagelkerke *R*^2^ = 0.142 indicating that the model explained approximately 14% of variance in turnover intention. These findings underscore the predominant role of organizational and structural factors in shaping midwives’ intentions to leave their positions.

### 3.7. Predictors of Job Performance

Hierarchical binary logistic regression analysis was conducted to examine predictors of job performance (good vs. moderate/poor), testing Hypothesis 3c. Variables were entered using forward stepwise (conditional) method from among demographic, organizational, and psychological resources variables.

The final model identified three significant predictors: overall self-leadership, natural reward strategies (a self-leadership subdimension), and number of children. Self-leadership total score emerged as a strong predictor of job performance (*B* = 0.915, *SE* = 0.286, *Wald* = 10.229, *p* = 0.001, *OR* = 2.497, 95% CI [1.426, 4.372]), indicating that each unit increase in self-leadership was associated with approximately 2.5 times higher odds of reporting good job performance. This represents a medium-to-large effect size and provides strong support for Hypothesis 3c.

Examination of self-leadership subdimensions revealed that natural reward strategies demonstrated a particularly robust association with job performance (*B* = 0.678, *SE* = 0.194, Wald = 12.224, *p* < 0.001, *OR* = 1.970, 95% CI [1.347, 2.882]). Each unit increase in natural reward strategies was associated with approximately twofold higher odds of good performance, suggesting that midwives who focus on the inherently rewarding aspects of their work are substantially more likely to perform at high levels.

Additionally, number of children showed a significant positive association with job performance (*B* = 0.336, SE = 0.162, *Wald* = 4.288, *p* = 0.038, *OR* = 1.399, 95% CI [1.019, 1.922]), with each additional child associated with approximately 40% higher odds of good performance. This unexpected finding may reflect increased efficiency, time management skills, or motivation among midwives with family responsibilities.

Notably, self-compassion—despite its theoretical role as a grounding spiritual and emotional resource—did not emerge as a significant predictor of job performance when self-leadership was included in the model (*p* > 0.05), suggesting that self-leadership captures the variance in performance more effectively than self-compassion. The final model demonstrated good fit (Hosmer–Lemeshow χ^2^(8) = 8.124, *p* = 0.421) and correctly classified 85.3% of cases, with Nagelkerke *R*^2^ = 0.187 indicating that the model explained approximately 19% of variance in job performance.

These findings provide strong support for Hypothesis 3c, demonstrating that self-leadership—particularly the natural reward strategies dimension—significantly predicts job performance among midwives, even when controlling for demographic and organizational factors.

### 3.8. Additional Findings

Work Experience and Psychological Distress: Although not part of the primary hypotheses, an exploratory analysis examined predictors of self-reported mental health diagnosis (present vs. absent) using binary logistic regression. Work experience emerged as a significant predictor of psychological distress (*B* = 0.070, *SE* = 0.024, *Wald* = 8.637, *p* = 0.003, *OR* = 1.073, 95% CI [1.024, 1.124]), indicating that each additional year of work experience was associated with approximately 7% higher odds of having received a mental health diagnosis. This finding suggests a cumulative burden effect, wherein prolonged exposure to the emotional and physical demands of midwifery practice may increase vulnerability to psychological distress over time. This pattern is consistent with compassion fatigue trajectories documented in previous research with Turkish midwives [6], which found that experienced midwives demonstrated increasing emotional detachment and professional indifference despite concurrent gains in clinical competence. The present finding extends this prior work by demonstrating a direct association between years of practice and formal mental health diagnoses, highlighting the need for targeted psychological support interventions for veteran midwives.

## 4. Discussion

This study investigated self-leadership and self-compassion as psychological resources among practicing midwives in Turkey, examining their associations with critical workforce outcomes including job satisfaction, turnover intention, and job performance. The research addressed four primary objectives: (1) determining the association between self-leadership and self-compassion, (2) examining demographic and organizational differences in these psychological resources, (3) testing their predictive roles in workforce outcomes, and (4) evaluating the relative contributions of organizational factors versus individual psychological resources in shaping professional experiences. Findings revealed a complex pattern of relationships that both confirm and challenge existing theoretical frameworks, with important implications for understanding midwifery workforce sustainability.

### 4.1. Self-Leadership and Self-Compassion an Integrated Resource Framework

Consistent with Hypothesis 1, self-leadership and self-compassion demonstrated a statistically significant positive correlation of moderate magnitude (r = 0.342, *p* < 0.01), indicating that midwives who reported higher self-leadership also tended to cultivate higher self-compassion. This suggests an integration of behavioral regulation with inner spiritual and emotional resources, supporting the conceptualization of these constructs as complementary rather than redundant psychological resources. This interpretation is consistent with theoretical perspectives suggesting that effective self-regulation encompasses both achievement-oriented strategies (self-leadership) and self-supportive emotional responses [34,40]. The moderate correlation magnitude indicates that while these resources share common variance—likely reflecting broader self-regulatory capacity—they remain sufficiently distinct to warrant separate consideration in workforce research and intervention design. Beyond its psychological regulatory role, self-compassion in this context may also reflect an underlying spiritual dimension [6,29,31]. By fostering a sense of common humanity and mindful awareness, self-compassion can connect midwives to the deeper, meaning-oriented aspects of their profession, aligning with the broader spiritual and meaning-oriented dimensions often discussed in holistic healthcare.

Consistent with this broader interpretation, the particularly strong association between behavior-focused self-leadership strategies and self-compassion (r = 0.355, *p* < 0.01) merits attention. Behavior-focused strategies, which include self-monitoring, self-reward, and self-punishment, represent concrete regulatory behaviors that directly shape performance patterns. The positive correlation with self-compassion suggests that midwives who actively monitor and regulate their behaviors may simultaneously develop greater self-awareness and self-kindness, consistent with mindfulness-based models of self-regulation [41]. However, the relatively modest correlation magnitude suggests these remain distinct processes, with self-compassion potentially serving as a protective factor against the potentially harsh self-evaluations that can accompany intensive self-monitoring.

This finding addresses the first research gap identified in the Introduction, wherein limited research has examined self-leadership and self-compassion together within healthcare contexts. The demonstration of their moderate positive association provides empirical support for integrated intervention approaches that simultaneously develop achievement-oriented self-regulatory skills and compassionate self-relating, rather than treating these as competing or incompatible orientations.

### 4.2. Demographic and Organizational Influences the Primacy of Education and Position

Hypothesis 2 received partial support, with self-leadership demonstrating significant variation according to educational level and professional position, while self-compassion showed no significant demographic or organizational differences. Self-leadership levels differed significantly by educational attainment (F = 5.125, *p* = 0.006), with master’s degree holders reporting higher self-leadership than those with bachelor’s or associate degrees. Similarly, professional position influenced self-leadership subdimensions, particularly natural reward strategies (F = 5.297, *p* = 0.005) and constructive thinking strategies (F = 3.119, *p* = 0.045), with team leaders and managers reporting higher levels than staff-level clinicians.

These patterns may reflect two complementary mechanisms that help explain the observed differences in self-leadership according to educational level and professional position. First, advanced education may directly cultivate self-leadership through exposure to leadership theories, reflective practice frameworks, and opportunities for autonomous decision-making within educational contexts [9]. Graduate-level midwifery education in Turkey increasingly emphasizes clinical leadership competencies, evidence-based practice, and professional autonomy, all of which align conceptually with self-leadership dimensions and may therefore contribute to the higher self-leadership levels observed among midwives with postgraduate education [11,42]. Second, selection effects may operate, suggesting that individuals with stronger pre-existing self-leadership tendencies may be more likely to pursue advanced education and leadership positions, thereby creating a reciprocal relationship between self-leadership and professional advancement [43].

The absence of demographic or organizational differences in self-compassion is noteworthy in contrast to the significant demographic variation observed in self-leadership and partially diverges from some healthcare literature suggesting that self-compassion may decrease with professional experience due to compassion fatigue [31]. Several interpretations may help explain this finding. Self-compassion may represent a more stable trait-like psychological characteristic less susceptible to environmental shaping than the skill-based components of self-leadership [30,44]. Alternatively, the cross-sectional design of the present study may obscure developmental trajectories, as self-compassion fluctuates across career stages but shows limited between-person variation at any single time point. Finally, cultural factors specific to Turkish midwifery contexts may influence how self-compassion is experienced or expressed, potentially differing from Western samples where most existing research has been conducted [29,35].

These findings address the second research gap regarding the limited understanding of how demographic and organizational factors shape psychological resources among midwives. The differential patterns for self-leadership versus self-compassion suggest that interventions targeting these resources may require distinct approaches: self-leadership development may benefit from integration into formal education and leadership development programs, while self-compassion cultivation may require universal approaches less dependent on organizational position or educational credentials.

### 4.3. The Organizational Context Paradox: When Psychological Resources Take a Backseat

Perhaps the most striking and theoretically challenging findings concern Hypotheses 3a and 3b, both of which were not supported regarding the predictive roles of self-leadership and self-compassion. Neither psychological resource significantly predicted job satisfaction or turnover intention when organizational factors were included in regression models. Instead, organizational variables—work schedule, healthcare setting, professional position, and income status—emerged as the primary predictors of these critical outcomes.

For job satisfaction, work schedule (β = 0.209, *p* < 0.001), healthcare setting (β = 0.199, *p* < 0.001), and professional position (β = 0.153, *p* = 0.004) collectively explained substantial variance, while self-leadership and self-compassion failed to demonstrate independent contributions. Similarly, for turnover intention, income status (χ^2^ = 10.357, *p* = 0.006), work schedule (χ^2^ = 7.469, *p* = 0.024), and job performance (OR = 0.512, *p* = 0.029) served as significant predictors, with psychological resources again showing no significant effects.

This pattern diverges from substantial prior research demonstrating that self-leadership predicts job satisfaction across diverse occupational contexts [10,26] and that self-compassion buffers against burnout and turnover intention among healthcare professionals [31,32]. In the present study, however, neither self-leadership nor self-compassion emerged as significant predictors of job satisfaction or turnover intention once organizational variables were included in the regression models. Instead, work schedule, healthcare setting, professional position, and income status appeared to play a more decisive role in shaping these outcomes. Nevertheless, this finding aligns with more recent international workforce sustainability research suggesting that organizational and structural conditions often exert stronger influence on workforce outcomes than individual psychological resources, particularly in resource-constrained healthcare systems [45,46]. In such contexts, structural features of the work environment may overshadow the potential benefits of individual-level psychological capacities.

Several contextual factors may help explain this pattern. The Turkish healthcare system has undergone substantial structural reforms in recent decades, resulting in work environments characterized by high patient loads, significant administrative demands, and ongoing resource constraints. These conditions are particularly evident in public hospitals, where the majority of participants in this study were employed [47,48,49]. Under such conditions, organizational factors may exert such powerful influence on satisfaction and retention that individual psychological resources become overwhelmed or rendered ineffective.

Second, the hierarchical regression approach employed may have created a particularly stringent test of psychological resource effects by entering organizational factors before psychological variables. This analytic strategy, while methodologically rigorous, may have obscured indirect effects wherein psychological resources influence satisfaction and turnover primarily through their impact on how individuals experience and respond to organizational conditions. Mediation analyses, not conducted in the present study, might reveal that self-leadership and self-compassion shape satisfaction and turnover indirectly by influencing perceived organizational support, work–life balance, or professional efficacy.

Third, cultural factors may moderate the salience of individual psychological resources versus organizational conditions. Turkish workplace culture emphasizes collectivism, hierarchy, and organizational loyalty [6] potentially rendering organizational factors more psychologically central than in individualistic Western contexts where much self-leadership and self-compassion research has been conducted [50]. In collectivist contexts, satisfaction and retention may depend more heavily on organizational functioning and relational dynamics than on individual self-regulatory capacities.

These unexpected findings address the third research gap regarding the limited understanding of how organizational factors interact with individual psychological resources in shaping midwifery workforce outcomes. The results suggest a more complex, context-dependent relationship than initially hypothesized, wherein organizational conditions may set boundary conditions within which psychological resources can operate effectively. This has important implications for intervention design: improving midwifery workforce sustainability may require addressing organizational structures and working conditions as a prerequisite for or complement to individual-level psychological interventions.

### 4.4. Self-Leadership and Performance: A Domain-Specific Success Story

In contrast to the null findings for satisfaction and turnover, Hypothesis 3c received strong support: self-leadership significantly predicted job performance (OR = 2.497, *p* = 0.001), representing a substantial effect wherein each unit increase in self-leadership was associated with approximately 2.5 times higher odds of reporting good performance. Natural reward strategies, a specific self-leadership subdimension focused on intrinsic motivation and finding inherent satisfaction in work tasks, demonstrated particularly robust effects (OR = 1.970, *p* < 0.001).

This pattern suggests a domain-specific model wherein self-leadership influences performance-related outcomes more strongly than affective or attitudinal outcomes. Several mechanisms may account for this specificity. Self-leadership strategies directly target goal-setting, self-monitoring, and task execution—processes immediately relevant to performance effectiveness. This may explain why self-leadership significantly predicted job performance in the present study, while showing no significant association with job satisfaction or turnover intention. In contrast, satisfaction and turnover intention reflect broader evaluations of work experiences influenced by numerous factors beyond individual task execution, including interpersonal relationships, organizational climate, compensation, and work–life balance [27,38].

The particularly strong effect of natural reward strategies aligns with self-determination theory [51], which posits that intrinsic motivation—finding inherent satisfaction and meaning in work activities—represents a more sustainable and powerful motivational force than extrinsic incentives. Midwives who actively cultivate awareness of and focus on the intrinsically rewarding aspects of their work—such as supporting women through childbirth, promoting family wellbeing, and contributing to positive health outcomes—may maintain higher performance levels even amid challenging organizational conditions. This finding has direct implications for performance management and professional development programs, suggesting that interventions emphasizing meaning-making and intrinsic motivation cultivation may yield stronger performance benefits than those focused solely on external rewards or punitive accountability.

The absence of self-compassion effects on performance, despite theoretical predictions that self-compassion should enhance performance by reducing performance anxiety and promoting adaptive responses to setbacks [34], warrants consideration. Self-compassion may influence performance primarily under conditions of failure, stress, or challenge—contexts where compassionate self-responding could prevent rumination and facilitate adaptive coping. The performance measure employed in this study assessed general self-rated performance rather than performance under specific stressful conditions, potentially obscuring conditional self-compassion effects. This distinction may also reflect the different functional roles of these psychological resources in clinical practice. Self-leadership primarily supports the behavioral execution of tasks and goal-directed performance [16,44]. By contrast, self-compassion may help preserve the emotional and spiritual capacity necessary for humanized care. In high-stakes clinical environments such as midwifery, the spiritual grounding associated with self-compassion may be reflected not primarily in technical performance indicators but in the quality of presence, empathy, and compassionate care provided to mothers. Additionally, cultural factors may moderate self-compassion’s performance relevance; in achievement-oriented cultures emphasizing self-discipline and effort, self-compassion might be perceived as incompatible with high performance, limiting its effectiveness even if theoretically beneficial.

### 4.5. The Experience Paradox: Accumulating Expertise, Declining Wellbeing

An exploratory finding revealed that work experience significantly predicted psychological distress, with each additional year of practice associated with approximately 7% higher odds of having received a mental health diagnosis (OR = 1.073, *p* = 0.003). This pattern is consistent with compassion fatigue models documented in Turkish midwifery contexts [6], which found that experienced midwives demonstrated increasing emotional detachment and professional indifference despite concurrent gains in clinical competence.

This experience paradox—wherein professional expertise accumulates alongside declining psychological wellbeing—likely reflects cumulative exposure to emotionally demanding situations characteristic of midwifery practice. Midwives routinely encounter pregnancy loss, neonatal complications, maternal morbidity, and family distress, all of which may contribute to a gradual accumulation of psychological strain over the course of a professional career [21,48,52]. Additionally, organizational factors may compound these individual-level stressors: experienced midwives often assume greater responsibilities, encounter more complex cases, and face intensified administrative burdens, while at the same time having limited access to structured psychological support or recovery opportunities within healthcare institutions [18,53].

The finding that work experience predicted psychological distress while self-compassion showed no significant demographic variation or protective effects against distress represents a troubling pattern suggesting that current levels of self-compassion among midwives may be insufficient to buffer against the cumulative psychological demands of practice. This underscores the need for proactive, sustained psychological support interventions targeting veteran practitioners, who may be at particular risk despite—or perhaps because of—their extensive experience and expertise.

The findings highlight an important distinction between proximal and distal outcomes in understanding the effects of individual psychological resources. Psychological resources, particularly self-leadership, demonstrated their strongest effects on proximal outcomes such as job performance, where self-leadership especially natural reward strategies, emerged as a robust and independent predictor. In contrast, more distal outcomes, including job satisfaction and turnover intention, were predominantly explained by organizational and structural factors (e.g., work schedule, healthcare setting, position, income), with psychological resources contributing little additional explanatory power once these contextual variables were considered. This pattern suggests that individual resources may exert their primary influence on immediate, task-related behaviors and self-regulatory processes, whereas broader attitudinal and behavioral intentions are more strongly shaped by external work conditions. Emphasizing this distinction helps contextualize the null findings for self-compassion and clarifies where individual psychological resources are most likely to be effective.

Our findings can be interpreted within established theoretical frameworks, including the Job Demands–Resources (JD-R) model and Conservation of Resources (COR) theory. Consistent with the JD-R model, individual psychological resources particularly self-leadership functioning as personal resources that enhance proximal outcomes such as job performance, especially when organizational demands are manageable. From a COR perspective, the limited impact of self-compassion on more distal outcomes, including job satisfaction and turnover intention, may reflect the predominant influence of organizational and structural resources, which buffer or exacerbate resource loss. Integrating these frameworks clarifies the mechanisms through which psychological resources operate and situates our results within established theoretical models, thereby strengthening the interpretive value of the study.

### 4.6. Theoretical Implications: Toward a Contextualized Model of Psychological Resources

Collectively, these findings necessitate refinement of existing theoretical frameworks regarding psychological resources in healthcare workforces. The results support a contextualized, domain-specific model wherein psychological resources demonstrate differential effectiveness depending on outcome domain and organizational context. Self-leadership appears particularly relevant for performance-related outcomes directly connected to task execution and goal achievement, while showing limited influence on affective outcomes (satisfaction) and behavioral intentions (turnover) that are more heavily shaped by organizational conditions.

This pattern suggests that psychological resources may function as “necessary but insufficient” conditions for positive workforce outcomes. Self-leadership and self-compassion may establish foundational capacities for effective functioning, but their translation into satisfaction and retention depends critically on whether organizational environments provide adequate support, resources, and working conditions. This perspective aligns with job demands–resources theory [54], which posits that personal resources interact with job resources to shape work outcomes, with personal resources showing stronger effects when job resources are adequate.

The differential findings for self-leadership versus self-compassion also suggest these constructs may operate through distinct pathways and demonstrate different contextual dependencies. Self-leadership, as an achievement-oriented resource focused on goal pursuit and task mastery, may influence outcomes through instrumental pathways—directly enhancing performance effectiveness and indirectly influencing satisfaction and retention through performance-based self-efficacy and accomplishment. In contrast, self-compassion, functioning as a psycho-spiritual resource focused on self-kindness, inner peace, and common humanity, may influence outcomes through both affective and spiritual pathways—buffering against distress and promoting deep-seated resilience under adversity. The present study’s design, emphasizing general outcome levels rather than responses to specific stressors or challenges, may have been better suited to detecting instrumental than affective pathways.

### 4.7. Practical Implications: A Multi-Level Intervention Framework

These findings have important implications for interventions aimed at midwifery workforce sustainability, underscoring the need for a multi-level approach that integrates individual psychological resources with organizational conditions. For enhancing job performance, individual-level interventions that strengthen self-leadership—particularly natural reward strategies focused on intrinsic motivation and meaning-making appear especially promising. Reflective practice, goal-setting and self-monitoring training, and mentorship programs in which experienced midwives model effective self-leadership may yield meaningful performance gains, as suggested by the substantial observed effect size. In addition to self-leadership development, complementary interventions targeting self-compassion may also be valuable. Specifically, structured self-compassion training [30,55] could be designed not only to reduce psychological distress but also to cultivate workplace spirituality, helping midwives reconnect with the fundamental meaning and humanistic values of their profession.

However, improving job satisfaction and reducing turnover intention require organizational-level interventions addressing workload, scheduling, compensation, and practice environments. Psychological interventions alone are unlikely to be effective if structural conditions remain suboptimal. Additionally, supporting experienced midwives calls for proactive psychological programs, such as structured self-compassion training combined with organizational supports including recovery time, peer networks, and professional recognition. Overall, these findings indicate that sustainable workforce strategies depend on integrating individual and organizational interventions, consistent with multi-level models of workforce sustainability [7].

### 4.8. Limitations and Directions for Future Research

Several limitations should be considered when interpreting these findings. The cross-sectional design precludes causal inference, leaving open the possibility of reverse or reciprocal relationships between self-leadership, self-compassion, and workforce outcomes; longitudinal designs are needed to clarify developmental trajectories and causal mechanisms. Reliance on self-report measures raises concerns about common method and social desirability bias, particularly for self-leadership and job performance. Although the Turkish adaptation of the Self-Compassion Scale demonstrated high reliability in previous studies [35], internal consistency in the current sample was moderate (α = 0.667), which may have attenuated observed associations. The 24-item length of the scale, along with several negatively worded items, likely contributed to the lower reliability. Despite this limitation, subscale reliabilities are reported to provide additional context for interpreting the effects of self-compassion in this study. A further limitation is that key variables, including job satisfaction, turnover intention, and job performance, were measured using single-item scales. Although single-item measures reduce participant burden and have been shown in recent research to exhibit reasonable validity and predictive performance comparable to multi-item measures in certain contexts (e.g., single items can correlate highly with corresponding multi-item constructs and predict related outcomes) [56,57], they may provide less nuanced measurement and could attenuate observed associations. In addition, convenience sampling through professional networks may limit generalizability, as the sample reflected relatively high educational attainment and low reported psychological distress.

Future studies should include more diverse samples across educational levels, work settings, and mental health profiles and be designed to support mediation and multilevel analyses to better examine potential indirect effects of psychological resources. The focus on general psychological resources rather than context-specific stressors may also have obscured conditional effects, suggesting value in experience-sampling or diary-based methodologies. Moreover, the Turkish cultural and healthcare context may constrain applicability to other settings, underscoring the need for cross-cultural replication. The future studies can be strengthened by considering cultural and situational conditions under which self-compassion may be more salient or effective. Cultural norms emphasizing collectivism, self-criticism, or emotional restraint, as well as situational factors such as acute versus chronic stress exposure, may moderate the effects of self-compassion and help explain the observed null findings. Future research should explicitly examine these cultural and contextual moderators, including cross-cultural comparisons and context-sensitive designs, to clarify when and for whom self-compassion is most effective.

Despite these limitations, the study makes several important contributions. It advances understanding by examining self-leadership and self-compassion jointly within midwifery practice, incorporating the role of workplace spirituality as a source of meaning, values alignment, and inner resilience, and applying rigorous hierarchical analyses to distinguish individual and organizational influences on workforce outcomes. By identifying outcome-specific patterns in the effectiveness of psychological and spiritual resources, these findings provide a foundation for more nuanced theoretical models and inform the development of targeted interventions aimed at strengthening inner resources and promoting the long-term sustainability of the midwifery workforce.

## 5. Conclusions

This study advances understanding of psychological resources and workforce sustainability in midwifery by jointly examining self-leadership and self-compassion within a broader framework that also considers the spiritual meaning often associated with midwifery practice, and by systematically comparing individual and organizational influences on key workforce outcomes. The findings reveal a context-dependent pattern: self-leadership particularly natural reward strategies emphasizing intrinsic motivation, meaning, and spiritual fulfillment derived from midwifery work strongly predicted job performance, whereas job satisfaction and turnover intention were primarily shaped by organizational factors such as work schedules, healthcare settings, professional roles, and income equity. Self-leadership, and self-compassion, were moderately interrelated yet demonstrated domain-specific effectiveness, challenging assumptions that psychological and meaning-related inner resources alone uniformly promote positive outcomes across workforce domains.

These results underscore the limits of individual-level interventions in the absence of supportive organizational conditions. While targeted self-leadership development may enhance performance, workforce sustainability depends largely on structural improvements addressing workload, scheduling, compensation, and practice environments especially for experienced midwives, who face increasing psychological strain over time. Within this context, psychological and spiritual dimensions of professional meaning may complement—but not replace—organizational supports. Overall, the study highlights the necessity of integrated, multi-level strategies that align psychological resource development with organizational reform to support midwives’ wellbeing, retention, and capacity to deliver high-quality care.

## Figures and Tables

**Table 1:** Sample characteristics.

Variable	Mean (SD) or Percentage (%)
**Age (Years)**	32.4249 ± 7.81239
**Work experience (years)**	10.0766 ± 8.74829
**Educational**	
HS/Associate	7.2
Bachelor’s	70.5
Master’s	22.3
**Income Status**	
Less than expenses	22.5
Equal	67.1
More	10.4
**Healthcare setting**	
Public	58.7
Private	19.7
Family health	17.1
University	4.6
**Position**	
Staff	81.5
Team leader	13.6
Manager	4.9
**Work schedule**	
Day	43.6
Night	15.0
Rotating	41.3
**Professional membership**	37.0
**Job satisfaction score**	8.260 ± 1.90
**Mental health diagnosis**	5.5
**Job Performance**	
Good	83.5
Moderate	16.5
**Turnover Intention**	
Present	31.5

**Table 2:** Descriptive Statistics and Internal Consistency Reliabilities for Self-Leadership and Self-Compassion Measures.

Variable	Mean	SD	Range	Skewness	Kurtosis	α
**SC Scale**	3.2531	1.9009	(2.17–5.00)	0.575	1.296	0.667
**SL Questionnaire (Total)**	4.0622	48.640	1.93–5.00	−0.930	1.587	0.90
Behavior-focused strategies	3.8663	53.204	1.75–5.00	−0.639	0.643	
*Reminders*	3.8540	1.04449	1.00–5.00	−0.849	0.059	0.794
*Self-rewarding*	3.9538	95.390	1.00–5.00	−0.932	0.513	0.877
*Self-punishment*	3.3714	87.667	1.25–5.00	−0.086	−0.624	0.765
*Self-monitoring*	4.2861	59.295	1.50–5.00	−0.985	1.548	0.737
Natural reward strategies	4.2919	69.682	1.00–5.00	−1.224	2.484	
*Thinking about natural rewards*	4.2919	69.682	1.00–5.00	−1.224	2.484	0.560
Constructive thinking model	4.1970	56.315	1.80–5.00	−0.849	1.276	
*Self-talk*	4.0568	76.370	1.33–5.00	−0.752	0.444	0.76
*Evaluating thoughts and ideas*	4.2962	57.643	2.00–5.00	−0.854	1.024	0.690
*Imagining successful performances*	4.2378	67.966	1.57–5.00	−1.086	1.245	0.884

**Table 3:** One-Way Analysis of Variance: Self-Leadership and Self-Compassion by Demographic and Organizational Characteristics. * *p* <0.1 ** *p* <0.05.

Variable	Educational Level		Income Status		Healthcare Setting		Work Schedule		Position	
	***F***	***p***	***F***	***p***	***F***	***p***	***F***	***p***	***F***	***p***
**SC Scale**	0.412	0.662	0.298	0.742	1.993	0.115	0.643	0.526	1.290	0.277
**SL Scale (Total)**	5.125	0.06 **	1.652	0.193	1.063	0.365	0.384	0.681	2.762	0.065 *
*Sub-dimensions:*										
Behavior-focused strategies	3.250	0.40 *	2.350	0.097 *	1.005	0.391	0.239	0.787	1.432	0.240
Natural reward strategies	6.030	0.03 **	2.382	0.094 *	0.630	0.596	0.062	0.939	5.297	0.005 **
Constructive thinking model	4.934	0.08 **	1.595	0.204	0.795	0.497	0.659	0.518	3.119	0.045 **

**Table 4:** Hierarchical Binary Logistic Regression Analysis: Predictors of Turnover Intention.

Predictor	*B*	*SE*	*Wald*	*df*	*p*	*OR*
**Income Status**			10.357	2	0.006	
Income < expenses	−0.496	0.285	3.042	1	0.081	0.609
Income = expenses	0.634	0.418	2.304	1	0.129	1.885
**Work Schedule**			7.469	2	0.024	
Permanent day shifts	0.843	0.349	5.823	1	0.016	2.323
Permanent night shift	0.572	0.264	4.690	1	0.030	1.772
**Job Performance** (Good performance)	−0.669	0.306	4.797	1	0.029	0.512
Constant	−0.363	0.361	1.009	1	0.315	0.696

## Data Availability

The data that support the findings of this study are available from the corresponding author upon reasonable request. The data are not publicly available due to ethical restrictions and the need to protect participant confidentiality.
